# Challenging Anaesthesia Management of a Patient with Fryns Syndrome: A Case Report

**DOI:** 10.4274/TJAR.2022.221038

**Published:** 2023-06-16

**Authors:** Celal Kaya, Pınar Kendigelen, Kadir Melih Yılmaz, Ayşe Çiğdem Tütüncü, Güner Kaya

**Affiliations:** 1 Department of Anaesthesiology and Intensive Care, İstanbul University-Cerrahpaşa, Cerrahpaşa Faculty of Medicine, İstanbul, Turkey

**Keywords:** Airway management, Fryns syndrome, pediatric anaesthesia, perioperative care, regional anaesthesia

## Abstract

Fryns syndrome cases with variable characteristics require careful preoperative evaluation and have challenges for airway management. Craniofacial anomalies can complicate both ventilation and intubation. Extubation can also be problematic because of limited pulmonary reserves.

**Main Points**

• Fryns syndrome with craniofacial anomalies should be regarded as candidates for difficult airway and preparations should be made accordingly.

• Anaesthesia and analgesia management must be done with utmost care and patients may require postoperative intensive care, especially for respiratory problems.

• Sugammadex as a reversal agent might be a good choice when there are concerns over airway management.

## Introduction

Fryns syndrome (FS) is a rare condition with multiple congenital malformations frequently including congenital diaphragmatic hernia (CDH), together with pulmonary hypoplasia, distal extremity hypoplasia, craniofacial, and internal anomalies.^1^ Although surgical interventions for CDH, gastrointestinal anomalies, and cleft palate are carried out in surviving infants, there are inadequate data on anaesthesia procedures for FS in the literature.^2^

## Case Presentation

**Figure 1:**
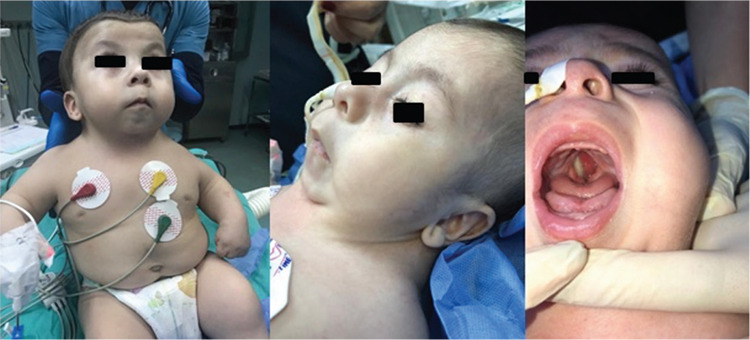
Fryns syndrome with short thick neck, micrognathia, coarse face, hypoplasic extremities and cleft palate.

Preoperative evaluation of the 16-month-old 9 kg male, who was in the 8^th^ percentile for weight and 2^nd^ percentile for height, with FS diagnosis showed typical features of coarse broad face, broad and flat nasal bridge, cleft palate, micrognathia, short and thick neck, widely spaced nipples, and hypoplastic extremities (Figure 1).

The patient, without a history of anaesthesia experience, was admitted for Nissen fundoplication following diagnosis of gastroesophageal reflux with frequent aspiration.

Preoperative echocardiography showed a thin (tubular) patent ductus arteriosus and focal septal hypertrophy. The results of laboratory tests were within normal limits. The patient’s heart rate (HR) was 145 min^-1^. There were secretory rales with lung auscultation due to chronic aspiration; his respiratory rate was around 50 min^-1^ and SpO_2_ was 96% in the room air.

Considering the patient’s neck and lower jaw structures, preparation for difficult intubation including laryngoscope blades, laryngeal masks, endotracheal tubes of various sizes, intubation stylets, and bougie endotracheal introducers was organised. Unfortunately, pediatric flexible fiberoptic bronchoscope was not available, so an ear-nose-throat surgeon was also called for an emergency.

First, a nasogastric tube was inserted and aspirated to avoid pulmonary aspiration, which was followed by anaesthesia induction with inhalational anaesthesia. Following successful face mask ventilation with 6% sevoflurane, a 26-gauge intravenous cannula was inserted and remifentanil (0.5 mg kg^-1^ min^-1^) infusion was commenced; finally, rocuronium (0.6 mg kg^-1^, IV) was administered. Neuromuscular monitoring was carried out with train of four (TOF) measurements. Before inhalational anaesthetic, the first TOF ratio was documented as 0.96 and “zero” TOF ratios was observed in the 55^th^ second after rocuronium. Afterwards, intubation was tried using the Macintosh laryngoscope size 1. During the first attempt neither the glottis nor the epiglottis could be seen, so Cormack Lehane classification^3^ was recorded as grade 4, but with the head in the sniffing position and cricoid pressure epiglottis and lower arytenoids were observed during second attempt and the trachea was intubated with the bougie, followed by sliding the 3.5 cuffed endotracheal tube over it. Next, the bougie was withdrawn, ventilation was commenced, and the endotracheal tube was fixed at 12 cm depth. Remifentanil infusion was decreased to 0.1 mg kg^-1^ min^-1^, sevoflurane was maintained at 2%, and IV dexamethasone (0.1 mg kg^-1^) was administered.

Ultrasound-guided central venous catheterization through the right internal jugular vein was performed. Subsequently, the distance between the skin and the dura was measured at the T10-11 level with ultrasound, which was approximately 18 mm. 6 mL of 0.125% bupivacaine was injected into epidural space with a 20 G Tuohy needle. The epidural space was reached at 15^th^ mm depth.

The total duration of surgery was 180 min. Perioperatively, HR was 100-140 min^-1^, mean blood pressure was 72-48 mmHg, SpO_2_ and EtCO_2_ ranges were 98-100% and 40-45 mmHg, respectively. Neuromuscular blockade monitoring was achieved and additional rocuronium (0.15 mg kg^-1^) was administered once during the operation. The patient without distinct haemorrhage was given 320 mL crystalloid intravenously and total diuresis was 50 mL. The operation ended without any complications.

Following 2 mg kg^-1^ IV sugammadex administration, 0.9 TOF ratio, which was used as a threshold for removing the intubation tube, was observed. Then, he was followed up in the recovery room for one hour using a modified Aldrete scoring system. Pain was monitored with visual analog scale score and additional analgesia was not needed after surgery even in the intensive care unit. Despite having an Aldrete score of 10, he was transferred to the pediatric intensive care unit for close monitoring and further treatment. He was transferred to the ward the day after surgery and discharged 2 days later.

## Discussion

Patients with FS present with differing clinical symptoms and anatomical defects.^1^ In cases indicated for surgery, complete cooperation with the surgical team is of primary importance. Preoperative anaesthesia examination must be performed with utmost care, evaluating all anomalies of the patient.

Given craniofacial malformations, the prospect of difficult ventilation and intubation should be kept in mind and preparations should be made accordingly.^4^ Despite being good options for difficult airways, video laryngoscopy and flexible fiberoptic bronchoscopy may not be available in many hospitals. Unluckily, we did not have them, so laryngoscope blades, laryngeal masks, endotracheal tubes of various sizes, intubation stylets, and bougie endotracheal introducers were prepared. At least two other experienced anaesthetists were also available during the procedure as recommended by studies in literature^4^. However, an ENT surgeon also presented for possible surgical airway in this particular case.

NMB monitoring was performed to make certain decisions about relaxation and complete reversal. The initial TOF ratio was recorded before induction to compare it after reversing with sugammadex. It has been shown that rocuronium-induced neuromuscular block can be fully reversed via sugammadex in a short time, which is comparable with succinylcholine; thus, it is possible to avoid succinylcholine-related side effects. However, there are many studies reporting the use of sugammadex in a “cannot ventilate and cannon intubate” situation.^5,6^

Intravenous cannulation can be difficult with hypoplastic extremities and hypotonia. A short and thick neck with limited extension can complicate central venous cannulation. Ultrasound guided catheterization is a safe approach as we have experienced.

Fluid therapy was maintained according to German guideline.^7^ A balanced isotonic electrolyte solution with 1% glucose was administered. The initial infusion rate was adjusted to 10 mL kg^-1^, then additional balanced isotonic solution was given due to patient loss from the nasogastric tube and surgical area. Fluid responsiveness was monitored with HR, blood pressure, skin turgor, venous blood gas analysis, and diuresis.

CDH is a significant cause of neonatal mortality and the associated pulmonary hypoplasia is frequently encountered in FS. Cases without CDH can present with diaphragmatic muscle weakness and elevated location of the diaphragm, which complicates respiration.^8^ In our patient, the diaphragm was elevated, causing tachypnoea and aspiration, which produced secretory rales. Therefore, during perioperative monitoring, possible mechanical complications were circumvented by avoiding high positive end-expiratory pressure and high tidal volume.

Providing effective analgesia is one of our main tasks to ensure rapid and comfortable postoperative recovery. Analgesia can be accomplished by a single dose epidural blockade in such patients.^9^ Opioid-spared analgesia was achieved using ultrasound-guided epidural bupivacaine to prevent nausea, vomiting, persistent sedation, and respiratory depression that complicate recovery, which has been a common practice of ours.

## Conclusion

Airway management can be challenging for syndromic patients with craniofacial anomalies. All necessary preparations must be done preoperatively. Opioid-spared analgesia achieved via regional techniques and complete antagonism with sugammadex for early extubation may prevent postoperative complications, enabling an early discharge from the hospital.
